# Emotional intelligence and happiness in clinical medical students: A cross‐sectional multicenter study

**DOI:** 10.1002/hsr2.1745

**Published:** 2023-12-08

**Authors:** Mohammad Reza Hatamnejad, Morteza Hosseinpour, Samin Shiati, Asal Seifaee, Mohammad Sayari, Faranak Seyyedi, Kamran Bagheri Lankarani, Sulmaz Ghahramani

**Affiliations:** ^1^ Health Policy Research Center, Institute of Health Shiraz University of Medical Sciences Shiraz Iran; ^2^ Student Research Department Shiraz University of Medical Sciences Shiraz Iran; ^3^ Department of Sociology Arak Azad University Arak Iran

**Keywords:** burnout, COVID‐19 pandemic, depersonalization, emotional intelligence, extraversion, happiness, medical student, perceived stress

## Abstract

**Background and Aims:**

Emotional intelligence (EI) has improved job performance and has been associated with the success of individuals. The interfering role of happiness in this equation is unneglectable; however, this association has not been established in clinical medical students exposed to work pressures and stressful hospital atmospheric. The present perusal was conducted to evaluate the relationship between EI and happiness in clinical medical students.

**Methods:**

A multicenter cross‐sectional investigation was conducted in Iran from December 2021 to June 2022. Multi‐stage cluster sampling followed by a systematic random selection was applied to choose participants. Data gathering was carried out online by Brief Emotional Intelligence Scale‐10 and Oxford Happiness Questionnaire.

**Results:**

Data from 405 participants were analyzed. The mean age was 24.2 years; men and women constituted 208 (51.4%) and 197 (48.6%) of all participants. Gamma regression analysis has determined age (Exp (B) = 1.016, *p*‐value < 0.001), female gender (Exp (B) = 0.966, *p*‐value = 0.04), extrovert personality (Exp (B) = 1.076, *p*‐value < 0.001), perceived somatic health (Exp (B) = 1.002, *p*‐value < 0.001), and stress level (Exp (B) = 0.990, *p*‐value = 0.01) as predictors of happiness. EI comprises five domains, and except for appraisal of others' emotions, an increase in all other domains of emotional intelligence was associated with higher happiness. GBRT model of significant variables revealed regulation of own emotions is the most happiness‐predictor variable (Exp (B) = 1.042, *p*‐value < 0.001).

**Conclusion:**

Diminishing the stresses helps medical students have a happier life. In happiness‐boosting strategies, vulnerable individuals (women, introverts, and those of higher age) ought to be noted as the targeted population. In this investigation, emotional intelligence was the most effective predictor of happiness in clinical medical students. Planning to promote happiness in these medical staff by enhancing their EI leads to better decision‐making and more prosperity in their job, resulting in better patient care services.

## 1 INTRODUCTION

In recent years, the topic of positive psychology, which deals with fields such as positive emotions, positive characteristics, and the role of these aspects in people's well‐being, has attracted the attention of many researchers.^1^ One of the essential parts of positive psychology is happiness, which means positive inner feelings in the face of events and, in general, a sense of life satisfaction.^2^ This element promotes physical and mental health and increases longevity.^3^ Happiness consists of emotional and cognitive components that can be affected by different personal and social factors such as socioeconomic and cultural status, living environment, and emotional intelligence (EI), possibly the most eminent.^4^

Salovey and Mayer initially defined EI as “The ability of individuals to understand and manage their own and others' feelings and use them to facilitate thinking.”^5^ It is directly associated with normal behaviors, good social interactions, and environmental adaptation; therefore, it can affect the individual's mental health.^6^, ^7^ Csikszentmihalyi figured out that happiness doesn't depend on external factors like money; instead, it is an inner feeling experienced when people are in a flow state. He described flow as a psychological state when people are completely absorbed into a challenging but possible task. They continue to do that like nothing else seems to matter, even if there is a remarkable cost to the seeker.^8^ The theory is postulated on different bases, for example, establishing a flow state needs immediate feedback as well as a balance between perceived challenge and skills to avoid anxiety or boredom.^9^ Proper feedback and perception come through how we intelligently appraise, regulate, and utilize emotion to know our capabilities and possible roadblocks. In other words, we are only enthusiastic about doing the works placed slightly above our skills, and challenging them brings us to a flow state and happiness, and this is feasible only if we are emotionally intelligent.^10^ As demonstrated by Lazzari et al.,^11^ this applies to healthcare undergraduate students. They experienced optimized interprofessional learning when there was an academic flow based on immediate reflection‐on‐action. They noticed their emotions via ecological momentary assessment (reflection) and learned how to react to them. Also, EI is demonstrated to be associated with some aspects of happiness, including positive moods, reducing negative thoughts, and healthy social relationships.^1^ Some studies suggest that people with higher EI have greater happiness and life satisfaction ^12^ and express higher levels of health as well.^13^

EI in medical education has been noticed recently, which has improved job performance and has been associated with the success of individuals.^14^ The interfering role of happiness in this equation is unneglectable; hence, by exploring the relationship between these two parameters in medical students, planning to promote EI ability and happiness level with the purpose of better decision‐making and being more prosperous in their job get possible.^15^

At first glance, it becomes apparent that EI can indirectly bring medical students happiness through diminishing elements like burnout^16^ and depression^17^ and boosting their mental health^18^ and life satisfaction.^19^ Blanchard et al.^16^ revealed higher EI level leads to reduced burnout in US medical students; Sfeir et al.^17^ emphasized the EI‐mediated association between extraversion and depression in Lebanese medical students; Khan et al.^18^ determined a meaningful direct correlation between EI and mental health in undergraduate medical students in western Uttar Pradesh. Ooi et al.^19^ stated that EI could positively predict life satisfaction among Malaysian undergraduates. However, through deeper scrutiny, some papers have mentioned the direct association between EI and happiness, whether in the medical industry or other jobs. Tiwari et al.^20^ declared EI can be associated with authentic happiness among Indians in late adolescence. Elayan et al.^21^ claimed that employer's EI could enhance employee happiness. Therefore, EI has been introduced by Fallahian et al.^22^ as a means of happiness mechanism in the workplace environment. In addition, this conclusion has been illustrated in healthcare professions. Ghajarzadeh et al.^14^ mentioned that happiness is a predictor of EI in medical residents in Iran. The predictability of happiness by EI is once more approved by the study of Gupta et al.^23^ on dental students of Faridabad. The significant association between EI and happiness in 120 medical students (not specified whether in basic science level or clinical grade) of Isfahan University of Medical Sciences (in Iran) was demonstrated by Sasanpour et al.^24^ Our previous study^25^ concluded a significant relationship between happiness and emotional intelligence in medical students at the basic science level; however, data on EI and happiness association in clinical medical students is missed. On the other hand, happiness and its influential factors differ between clinical and basic science medical learners, as clinical students are exposed to more stressful events, including committing a medical fault, observing the patient's expiration, and upcoming residency exams. In this context, Kulkarni et al.^26^ revealed that happiness level decreases marginally as the academic year progresses. Thus, contrary to our previous research,^25^ in which such a relation was scrutinized in preclinical medical students, medical students at the clinical stage are now targeted.

Medical education in Iran is a 7‐year (14 semesters) program with preclinical and clinical phases. The preclinical course comprises 2.5 years (five semesters) of studying the basic science programs and 1 year (two semesters) of undertaking the Physiopathology stage. The clinical practice consists of first‐year Stager, second‐year Stager, and internship. First and second‐year Stagers are equivalent to the first and second‐year medical clerkships in other medical educations. The internship is also assumed as a postgraduation program in different medical education systems, but in Iran, it is assumed as part of pre‐graduation curriculum. Hence, Stager and Intern grades are considered to appraise the study purpose within the clinical medical students.

Overworking pressures and stressful hospital atmospheres, particularly with emerging situations like the COVID‐19 pandemic, affect the students' happiness level and their clinical practice.^27^ Therefore, it is more reasonable to consider these junior medical staff (interns and stagers) to boost the quality of patient care and clinical performance. So, this study has been performed to evaluate the relationship between EI and happiness in Stager and Intern medical students. We hypothesized that EI is significantly correlated with happiness levels among clinical medical students, and independent variables affecting this association differ from the previous investigation, which was conducted in the setting of medical students who have not experienced clinical practice.^25^

## 2 MATERIALS AND METHODS

### 2.1 Study design

The current cross‐sectional, observational, and multicenter investigation was conducted at medical colleges of the principal universities of Fars province in Iran, including Shiraz, Fasa, and Jahrom University of Medical Sciences, from December 2021 to June 2022. The study was performed in compliance with the international guidelines on clinical investigation of the World Medical Association's Declaration of Helsinki; the Shiraz University ethics committee approved the study protocol (ethics code: IR. SUMS. REC.1400.828).

### 2.2 Aims and objectives

#### 2.2.1 Study aims

Evaluation of EI and happiness association in clinical medical students.

**Research hypothesis (H**_**1**_ **):** EI and happiness significantly correlate in clinical medical students.

**Null hypothesis (H**_**0**_ **):** No significant correlation exists between EI and happiness in clinical medical students.

#### 2.2.2 Objectives

− Determining the total happiness score in clinical medical students.
− Determining the total emotional intelligence and its domains scores in clinical medical students.
− Determining the predictors (independent variables) of happiness (dependent variable) in clinical medical students.
− Evaluation of EI and its domains as predictors of happiness in the targeted population.

### 2.3 Participants selection

The minimum sample size, using Cochran's formula, with the predicted lowest correlation coefficient of 0.25, 95% confidence interval, 5% alpha error, and 80% power, was estimated at 123 students; however, to conduct the study in the three centers, the engagement cut‐off point was considered as 400 contributors.

**Figure 1:**
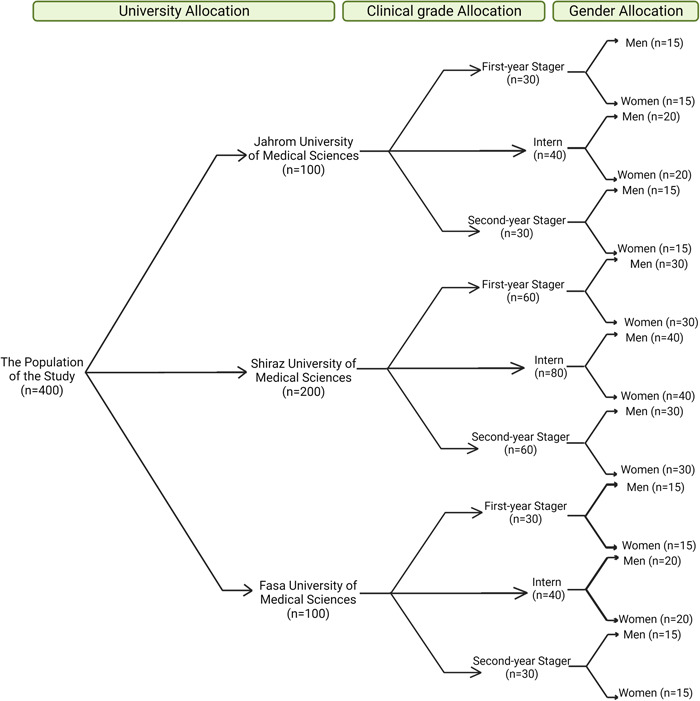
Multi‐stage cluster sampling. At first, the allocated contributing proportion to each university and clinical stage was determined based on its population; therefore, participants were dedicated to Shiraz University of Medical Sciences, and intern grades two times higher than other sub‐classes. Then, attempts were made to have almost equal numbers of men and women participants.

Multi‐stage cluster sampling was performed in three steps (Figure 1). At first, the allocated contributing proportion to each university and the clinical stage was determined based on its population; therefore, participants were dedicated to Shiraz University of Medical Sciences, and intern grades were two times higher than other sub‐classes. Then, attempts were made to have almost equal numbers of men and women participants. Systematic random sampling was exerted to select the participants within each cluster. Willing candidates were included based on providing written informed consent and not being a guest or exchanged student. The exclusion was considered for those who filled out the questionnaires incompletely.

**Figure 2:**
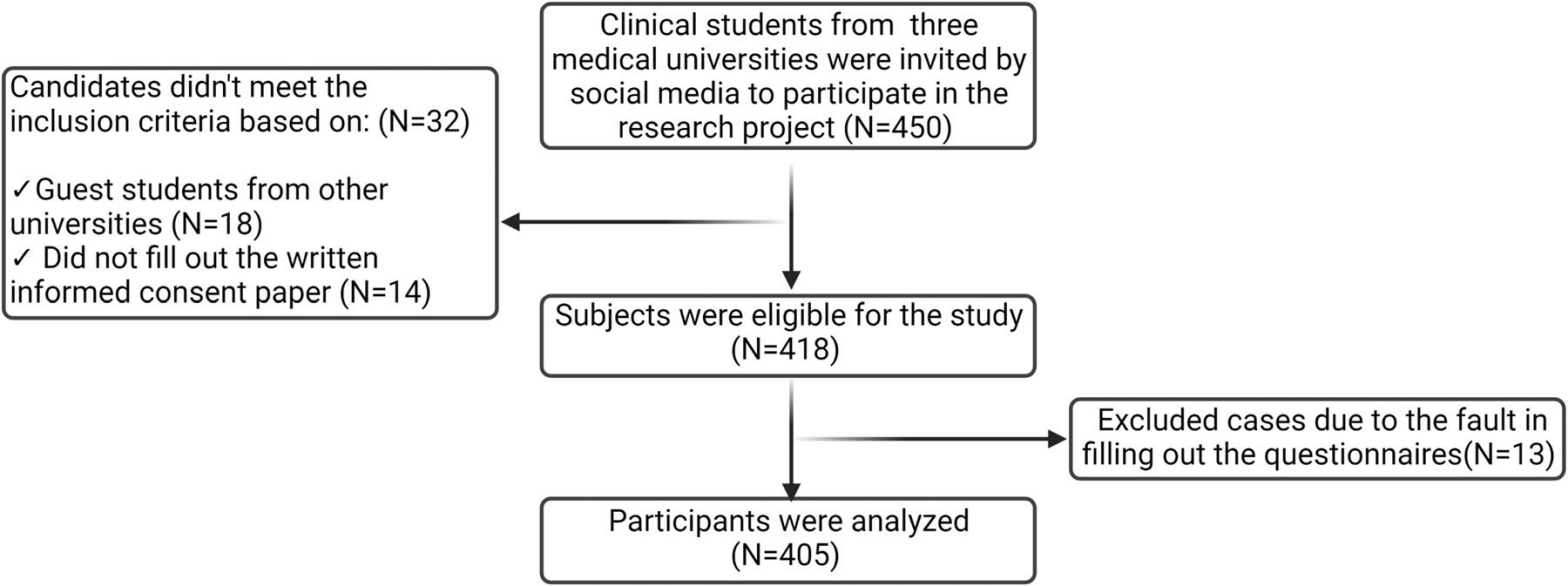
Flowchart of participant's selection. Among 450 clinical students who were invited by social media to participate in the research project, 418 were eligible for the study. Thirteen students were excluded. Thus, the data of 405 participants were analyzed.

The flowchart of the participant's selection is presented in Figure 2. During the study period, 450 clinical students from three medical universities were invited to participate in the research project. Among them, 32 students did not meet the inclusion criteria; thus, 418 students were eligible for the study. Thirteen students were excluded after the primary assessment due to incomplete filling out the questionnaires. Therefore, the data of 405 students were analyzed.

### 2.4 Data gathering process

Due to the limitations imposed by the COVID‐19 pandemic, the data gathering was mainly carried out online via social networks including, WhatsApp, Telegram, and electronic email. Individuals were contacted, and an informed consent form was sent. After explaining the purposes of the research and obtaining their agreement, a link to accomplish the questionnaire was given.

### 2.5 Data collection instruments

The questionnaire contains three main parts. Information on various participants' backgrounds, including age, gender, educational level, socioeconomic status, family structure, and personal self‐evaluations, constitutes the demographic component.

In personal self‐evaluation, participants were asked about happiness‐affecting factors, including depersonalization,^28^ emotional exhaustion (burnout),^29^ and perceived stress/health status in the last 60 days. EI comprises the appraisal and regulation of one's emotions; thereby, more intelligently use of emotions leads to more awareness about the stress/health status and more efficient adjusting them to come up with happiness and well‐being.^30^ Literature addressed the interrelational role of EI in the perception of stress/health and happiness. Ruiz‐Arandain et al. pointed out the mediating effect of perceived stress in the relation between happiness and EI in female student health professionals.^31^ Augusto Landa et al. explained that EI leads to individual stress‐handling disparities, eventually resulting in different happiness statuses.^32^ In addition, a significant effect of perceived stress/health status was discovered in the previous paper about EI and happiness in medical students at the basic science level^25^; hence, perceived stress and health status were assessed by a visual analog scale (VAS), resembling our previous study. In this method, participants were requested to estimate their perceived stress/health statuses by donating a number from 0 to 10 (for stress) or 100 (for health). Exploitation of VAS to assess perceived health and stress levels was previously demonstrated by Nicholas^33^ and Lesage et al.,^34^ respectively. Besides, depersonalization, emotional exhaustion, the detrimental impact of COVID‐19 development,^35^ and thinking about forced international migration and its negative effect on happiness,^36^ as the other self‐perceptive questions, were assessed qualitatively (First, they read what the feature [e.g., depersonalization] means and then check whether this event happened to them [existed or not]).

In subsequent sections, measuring emotional intelligence and happiness was facilitated by the Brief Emotional Intelligence Scale‐10 (BEIS‐10) and Oxford Happiness Questionnaire, respectively.

#### 2.5.1 Brief Emotional Intelligence Scale‐10

As a shortened version of the Schutte 33‐item Emotional Intelligence Scale,^37^ Davies et al. developed a valid brief questionnaire containing five domains: appraisal of own emotions, regulation of own emotions, appraisal of others' emotions, regulation of others' emotions, and utilization of emotions.^38^ Each domain comprises two sectors that subjects can answer with a 5‐point Likert scale from 1 (never) to 5 (always). Eventually, the total score of the 10‐item questionnaire is from 10 to 50, and individuals with higher scores have more EI ability. Hadadian et al.^39^ explored trans‐cultural adaptation in Iranian society and represented that the Persian version of BEIS‐10 is valid and reliable (intraclass correlation coefficient= 0.612, Cronbach's alpha = 0.748). Respecting the briefness and time‐saving properties of BIES‐10 compared with other EI assessment scales, the investigators utilized the BEIS‐10 in contrast to the prior experience that the Siberia Schering questionnaire employed.^25^

#### 2.5.2 Oxford happiness questionnaire

Argyle and Hills extended the range of answering Likert score (from 4 to 6 degrees), applied the reverse scoring for 12 questions with the inverted verb, and ultimately established the Oxford happiness questionnaire to enhance the accuracy of the previous version of the happiness questionnaire (Oxford happiness Inventory).^40^ It is organized by 29 items related to seven domains: self‐concept, aesthetic feeling, self‐efficacy, emotional readiness, life satisfaction, hopefulness, and spiritual intelligence. Each person can acquire a score from 29 to 174, divided by 29 to calculate the happiness score; higher marks indicate happier ones. Najafi et al. confirmed the reliability and validity of the questionnaire, and Cronbach's α was calculated at 0.901.^41^

### 2.6 Statistical analysis

Mean ± standard deviation was used to express continuous variables. However, categorical variables were represented by numbers and percentages. This research used a two‐stage procedure to evaluate the effects of variables related to happiness. In the first stage, gamma regression was used to identify the significant variables. The gamma regression with a log link is suitable for analyzing positively skewed continuous outcomes. In the second stage, the significant variables were entered into the gradient‐boosted regression trees (GBRT) model to extract the variable's importance. As a machine‐learning method, GBRT modifies the classification and regression trees. GBRT generates regression trees sequentially, drawing upon the information from previous trees to enhance prediction performance. The final model results from aggregating weak prediction models (learners).^42^, ^43^ The statistical analysis, including gamma regression, was carried out using Stata (version 17.0); the GBRT procedure was performed via the R statistical package (version 4.1.1). A *p*‐value less than 0.05 considers the result substantial.

## 3 RESULTS

The detail of the data is illustrated in Table 1. The mean age was 24.2 years; men and women constituted 208 (51.4%) and 197 (48.6%) of all participants.

**Table 1:** Descriptive statistics of participants' variables

Variables	Mean ± SD or Number (%)	Variables	Mean ± SD or Number (%)
**Age**	24.2 ± 1.82	**Father's Job**	
**Gender**		Employee	362 (89.4%)
Men	208 (51.4%)	Unemployed	13 (3.2%)
Women	197 (48.6%)	Retired with pension	30 (7.4%)
**Relationship Status**		**Father's educational level**	
Single	254 (62.7%)	Illiterate	8 (2%)
Married	54 (13.3%)	Middle school diploma	22 (5.4%)
In a relationship	97 (24%)	High school diploma	111 (27.4%)
**Clinical Grade**		University's degrees	264 (65.2%)
First‐year Stager	108 (26.7%)	**Mother's educational level**	
Second‐year Stager	116 (28.6%)	Illiterate	17 (4.2%)
Intern	181 (44.7%)	Middle school diploma	41 (10.1%)
**Tuition status**		High school diploma	143 (35.3%)
Scholarships students	329 (81.2%)	University's degrees	204 (50.4%)
Fee‐paying students	76 (18.8%)	**Parental Living Status**	
**University**		Joint living parents	344 (84.9%)
Shiraz	203 (50.1%)	Separated parents	22 (5.4%)
Fasa	100 (24.7%)	Deceased a parent	39 (9.6%)
Jahrom	102 (25.2%)	**Self‐perception** b	
**Place of Living** a		Stress level^c^	5.82 ± 2.31
Dormitory	163 (40.2%)	Somatic health level^c^	81.8 ± 16.6
Family residence	135 (33.3%)	Depersonalization^d^	162 (40%)
Independent residence	107 (26.4%)	Believed in negative COVID‐19 impact on happiness	241 (59.5%)
**Ethnicity**			
Persian	262 (64.7%)		
Others	143 (35.3%)	Emotional exhaustion Thinking about migrating **Emotional Intelligence** e	271 (66.9%) 262 (64.7%)
**Students with a part‐time job**	93 (23%)		
**Salary**			
Without Income	183 (45.1%)	Appraisal of own emotions	6.94 ± 1.52
With Income	222 (54.9%)	Regulation of own emotions	6.98 ± 1.50
**Income‐expenses Balance**		Appraisal of others' emotions	6.76 ± 1.64
Expenses > Income	159 (39.3%)	Regulation others' emotions	6.61 ± 1.64
Expenses = Income	194 (47.9%)	Utilization of emotions	7.38 ± 1.56
Expenses < Income	52 (12.8%)	Total score (10–50)	34.6 ± 6.16
**Siblings**		**Happiness scores (Total range/Number of**	
One and two	283 (69.9%)	**items)** f	
Three and more	122 (30.1%)	Self‐concept (8–48)/8	3.74 ± 1.05
**Special talent's high‐schools**	267 (65.9%)	Aesthetic Feeling (5–30)/5	3.16 ± 0.78
**Top student of class (rank 1**–**10)**	69 (17%)	Self‐efficacy (5–30)/5	3.25 ± 0.53
**Personality**		Emotional Readiness (4–24)/4	3.77 ± 1.05
Introvert	233 (57.5%)	Life satisfaction (3–18)/3	3.58 ± 1.25
Extrovert	172 (42.5%)	Hopefulness (2–12)/2	3.94 ± 1.23
**Mother's Job**		Spiritual Intelligence (2–12)/2	3.71 ± 1.26
Housekeeper	230 (56.8%)	Mean total Score (29–174)/29	3.78 ± 0.85
Employee	175 (43.2%)		

^a^ Family residence means living with other family members in a shared house; Independent residence means living apart from family in a separate home.

^b^ Self‐perception refers to the personal self‐evaluations within the last 60‐days.

^c^ Perceived stress and health status were assessed by a visual analog scale. In this method, participants were requested to estimate their perceived stress/health statuses by donating a number from 0 to 10 (for stress) or 100 (for health).

^d^ It defines as: I get the feeling that I treat some patients/colleagues impersonally as if they were objects.

^e^ The Emotional Intelligence domains' scores range are 2–10.

^f^ The mean scores of happiness and its domains' range are 1–6.

The majority of subjects believed in (1) the adverse impression of COVID‐19 on their happiness level 241 (59.5%), (2) emotional exhaustion about hospital work 271 (66.9%), (3) and forced immigration to developed countries to find a job or proceed the education 262 (64.7%). Mostly, they assumed themselves as introverted individuals 233 (57.5%), with a relatively healthy physical status (mean estimated score of 81.8 on a scale from 0 to 100) and a moderately stressful life (mean estimated score of 5.82 on a scale from 0 to 10). EI (34.6/50, 69.2% of total) and mean happiness (3.78/6, 63% of total) scores indicate research candidates are placed in the second quartile and possess relatively sufficient happiness and EI. Inquiring into each category domain demonstrates the utilization of emotions (7.38/10), and hopefulness (3.94/6) are the categories related to EI and happiness, respectively, that students are most efficient.

Gamma regression analysis of happiness and its independent variables are shown in Table 2. Compared to men, women significantly owned lower happiness (Exp (B) = 0.966). Individuals with extroverted personalities compared to introverted ones significantly possessed higher happiness (Exp (B) = 1.076). In addition, increasing age (Exp (B) = 1.016 per year) and somatic (physical) health level (Exp (B) = 1.002 per one score) have been found to be significant predictors of happiness. On the contrary, a significant reverse happiness level was obtained in the individuals with higher stress levels (Exp (B) = 0.990, per one score). Except for the appraisal of others' emotions, an increase in all other domains of emotional intelligence was associated with higher happiness (H_1_ was approved, and H_0_ was declined).

**Table 2:** Gamma regression analysis of happiness and its independent variables.

Categorical variables (constant values)^a^		Exp (B)	Std. Err.	P > Z	95% Confidence Interval
**Gender (Men)**					
Women		0.966	0.016	**0.037**	0.935–0.998
**Relationship Status (Single)**					
Married		1.026	0.026	0.318	0.976–1.078
In a relationship		1.009	0.020	0.668	0.969–1.050
**Clinical grade (First‐year Stager)**					
Two‐year stager		1.024	0.024	0.320	0.977–1.072
Intern		1.017	0.023	0.467	0.973–1.063
**University (Shiraz)**					
Fasa		1.005	0.021	0.819	0.964–1.047
Jahrom		1.023	0.021	0.275	0.982–1.065
**Place of Living (Dormitory)**					
Family residence		1.025	0.021	0.222	0.985–1.068
Independent residence		1.037	0.021	0.076	0.996–1.080
**Ethnicity (Persian)**					
Others		1.033	0.019	0.077	0.966–1.070
**A part‐time job (Without)**					
Own a part‐time job		1.025	0.023	0.288	0.980–1.072
**Salary (Without Income)**					
With Income		0.982	0.020	0.370	0.944–1.022
**Siblings (One and two)**					
Three and more Siblings		0.983	0.018	0.363	0.944–1.022
**Top student of class (Not being)**					
Being a top student of class		1.020	0.022	0.370	0.977–1.064
**Parental Living Status (Joint living parents)**					
Separated Parents		0.976	0.035	0.506	0.909–1.048
Deceased a parent		1.013	0.028	0.635	0.960–1.070
**Personality (Introvert)**					
Extrovert		1.076	0.018	**0.000**	1.041–1.112
**Forced migrating and its negative impacts (Not thinking)**					
Thinking about migrating		0.967	0.017	0.052	0.935–1.000
**Emotional exhaustion (Without)**					
Emotionally exhausted		0.977	0.018	0.205	0.942–1.013
**Depersonalization (Without)**					
Depersonalized		0.968	0.017	0.061	0.935–1.002
**Happiness compared before pandemic (Get worse)**					
Get better		1.024	0.018	0.165	0.990–1.059
Continuous variables		Exp (B)	Std. Err.	P > Z	95% Confidence Interval
**Age**		1.016	0.003	**0.000**	1.010–1.022
**Stress level perception**		0.990	0.004	**0.008**	0.982–0.997
**Somatic health level perception**		1.002	0.000	**0.000**	1.001–1.003
**EI**	Appraisal of own emotions	1.023	0.008	**0.002**	1.009–1.038
	Regulation of own emotions	1.042	0.008	**0.000**	1.027–1.058
	Appraisal of others’ emotions	0.995	0.007	0.493	0.982–1.009
	Regulation others’ emotions	1.026	0.007	**0.000**	1.014–1.039
	Utilization of emotions	1.030	0.007	**0.000**	1.016–1.045

^a^ Constant values were considered as the base class for each category regression analysis.

**Figure 3:**
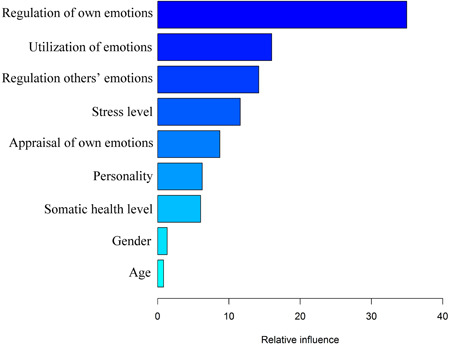
GBRT to indicate the impact of the variables in predicting happiness.

The result of GBRT in terms of relative influence, which shows the impact of the explanatory variables in predicting happiness, is illustrated in Figure 3. As can be seen, regulation of own emotions is the most happiness‐predictor variable (Exp (B) = 1.042, *p*‐value < 0.001), followed by utilization of emotions (Exp (B) = 1.030, *p*‐value < 0.001).

## 4 DISCUSSION

Happiness positively and significantly affects performance in the workplace,^44^ so it can lead to the better function of clinical students and improve the care of hospitalized patients. Various factors affect happiness. EI is one of the paramount happiness‐facilitating elements.^2^ The present study evaluated the relationship between happiness and EI in medical students at the clinical stage, which showed a significant positive correlation between these variables. The results also support the relation of happiness with age, gender, personality, and self‐perceptions about somatic health and stress level.

The limited literature on happiness and EI relation in medical education made our work distinctive.^4^, ^45^ Moreover, it is noteworthy to mention that current research differs from previous projects regarding the use of BEIS‐10 as an EI indicator ^25^, ^46^, ^47^, ^48^ and large sample size (multicenter methodology).^4^, ^16^, ^49^ Besides, more comparative detail will be elaborated between present and previous studies to clarify the study methodology and justification for conducting a similar project. Firstly, in the current investigation, the Oxford Happiness Questionnaire was applied. It comprises a broader Likert range of answers with a reverse scoring system that, in comparison to the previous study in which the Oxford Happiness Inventory was used, resulted in a more accurate happiness measurement. Also, BIES‐10 aided us in scaling the EI and led to a higher response rate than the previous study in which a 33‐item Siberia Schering questionnaire had been employed. Secondly, the current investigation was conducted on 405 subjects in three medical centers; meanwhile, the former perusal explored the research question in 300 participants in one medical university. Thirdly, two study designs measured different confounding factors in EI and happiness association. The former project appraised age, gender, perceived stress/health status, personality, and educational grade along with EI as the predictors of happiness; nevertheless, present research considered more factors, like sibling, ethnicity, place of living, parental socio‐educational status, living expenses, and perceived burnout/depersonalization feeling in addition to previous elements for happiness evaluation; all these aspects narrate generalizability of the current study result.

Happiness showed a negative statistically significant relationship with aging in this perusal. Blanchflower^50^ re‐examined the relationship between various measures of well‐being and happiness with age in 145 countries, confirming the U‐shape curve relation between these variables. It mentioned that the nadir part of the curve is located around midlife, which can be due to exposure to life problems. At this stage of life, people need to undertake responsibility. The consequences of confronting multiple stressors at this stage can stop or even decrease life satisfaction until the brain adapts to the new situation, learns, and allows life satisfaction to increase again.^51^ However, Steptoe et al.^52^ believed that the U‐shape pattern is not universal and differs in some parts of the world. Frackowiak et al.^53^ showed age as a negative predictor of happiness among Polish people. As mentioned in this study, a negative correlation between age and happiness may be due to the participants' age range located in descending part of the U‐shape curve.

In the current study, happiness showed a positive correlation with the male gender, which contrasts with Meimanat's^54^ essay. Mahmoodi et al. found that the men students were happier compared to women. These differences may be attributed to social context and cultural norms that may cause gender‐based differences.^55^ These differences can be attributed to gender‐related roles (such as childbearing, maternal responsibilities, employment, and parenthood) and social support (i.e., emotional, financial, and instrumental), which lead to the diversity in gender base individual happiness.^56^

Extraversion is an energetic approach to social life that includes socialization, activeness, resoluteness, and courage. Personality evaluations represented that introverts encounter less pleasure and more pain,^57^ and on the other hand, researchers revealed that extroverts are happier^57^, ^58^, ^59^ based on different grounds. Affective reactivity is one of the hypotheses that may justify the fact that extroverts are happier. It explains in response to positive or rewarding stimuli (situations where the person receives a reward in exchange for accomplishing a task), extroversions reflect more positive satisfaction, such as happiness.^60^ Another explaining theory is the Person‐by‐Situation Fit perspective. The group participating in activities is much more gratifying than individually performing; consequently, extroverts with more social interaction experience more happiness. Hence, the direct effect of extroversion on momentary pleasure will be mediated by rewarding activities and social interactions.^61^ Richițeanu‐Năstase et al. believed that extroverts are happier; nevertheless, education can alter this personality component. They suggested workshops and counseling centers should be held by universities to make students more joyful, social, and efficient.^58^ Lauriola et al. believed that extroverts are happier than others because they approach life positively and with greater confidence.^62^ However, the present paper, aligned with the previous investigation,^25^ suggests extrovert personality is a potent indicator in every domain of happiness.

This investigation showed that a less stressful life connects with happiness. Dhingra et al. indicated that a high level of perceived stress leads to a feeling of low psychological well‐being and happiness.^63^ Mehrzad et al. demonstrated that happiness has a reverse relationship with depression, anxiety, and stress. Stress leads to emotional imbalance and physiological changes in the body, interfering with vital processes and affecting mental health and happiness.^64^ Csikszentmihalyi explained workflow as an optimal state in which persons eagerly participate in activities regardless of their consequences^8^, ^10^; on this occasion, there is a direct balance between our skills and challenges to be in a flow state. If our skills exceed the challenge's requirement, we will get bored; however, overwhelming the challenge demands on our capabilities makes us stressed and anxious.^9^ Therefore, skill‐boosting educational strategies are necessary to restore the mentioned balance; Lazzari et al., throughout repetitive real‐time data gathering via an ecological momentary survey, figured out that undergraduate healthcare students were more satisfied and with lower stress when they underwent interprofessional education courses, accompanied by more patient care and less fault.^11^

A link between three areas of EI, well‐being (happiness), and career success has been illustrated before.^65^ The investigation by Badri and his colleagues on young adults demonstrated a robust statistical relationship between EI and happiness.^4^ Callea et al. conducted a study on 216 participants; They revealed that people with higher levels of EI own more elevated levels of flourishing and happiness.^13^ Extremera et al. disclosed that coping ability, focus on planning, positive reappraisal, and putting into perspective are bridging pathways between EI, psychological well‐being, and life satisfaction.^66^ Present perusal confirmed the premise of parallel association between these factors and is consistent with our previous scrutiny.^25^

In a systematic review, Kotsou et al.^67^ demonstrated that among 46 interventional studies for enhancing EI, two of them were dedicated to medical students; meanwhile, four investigations were conducted on the other medical staff. The core of these interventions was postulated based on training; however, different approaches have led to different outcomes. Abe et al.^68^ held a 2‐h mental health workshop for medical students, and at the end of the intervention, EI didn't change significantly. In contrast, Dugan et al.^69^ demonstrated that with an 8‐h highly interactive EI training within 7 years, a significant change in EI level appears to correlate with patient satisfaction. In addition, Fletcher et al.^70^ concluded that 4‐h EI workshops within a 7‐month training program effectively enhance EI in medical students. Therefore, pooled results suggest training workshops (especially in concise sessions within the long interventional period) as the EI boosting strategies in medical students at clinical grade. Planning to promote happiness in these medical staff by enhancing their EI leads to better decision‐making and more prosperity in their job, which subsequently results in better patient care services.

The cross‐sectional nature of the study design limits the causality claim for the association between EI and happiness and could be better validated through a cohort study; thus, further studies are required. Evaluation of depersonalization and emotional exhaustion in the workplace (burnout) were not carried out by their specific questionnaire (such as the Burnout Assessment Tool or Maslach Burnout Inventory) since the present data questionnaire is lengthy (80 items), and if more questions were added, it would be tedious and may cause the failure to fill out the entire questionnaire (decreasing the response rate); therefore, they were evaluated in self‐perception qualitative mode (First, they read what the feature [e.g., depersonalization] means and then check whether this event happened to them [existed or not] in previous 60 days). This may have affected our result; nevertheless, all efforts were made to alleviate this limitation by evaluating their qualitative values. Yildirim et al.^71^ exhibited that levels of perceived risk associated with COVID‐19 can harm mental health by stimulating morbidity susceptibility, reducing happiness, and diminishing positive views about self, life, and the future. In addition, COVID‐19, through causing burnout among health workers, alleviated happiness levels^72^, ^73^; although, in this investigation, the impact of the COVID‐19 pandemic on happiness was not proved. Such a result may have arisen due to the cross‐sectional methodology limitation; long period follow‐up for a more precise conclusion is needed.

## 5 CONCLUSION

Present perusal introduced EI, age, gender, personality, and self‐perception (about somatic health and stress level) as independent predictors of happiness in clinical medical students. These outcomes convey some concepts. Diminishing the stresses in life approaches could help medical students to have happier lives. Women, introverts, and those of higher age are more prone to lower happiness levels; hence, these vulnerable individuals should be noted as the targeted population in happiness‐boosting strategies. Further studies, with long‐term follow‐up (cohort setting) and more comprehensive population study (participants from all medical stages), are recommended to explore the association of EI and happiness more accurately.

## AUTHOR CONTRIBUTIONS

**Mohammad Reza Hatamnejad**: Conceptualization; Data curation; Formal analysis; Investigation; Methodology; Resources; Software; Validation; Visualization; Writing—original draft; Writing—review & editing. **Morteza Hosseinpour**: Investigation; Methodology; Writing—original draft; Writing—review & editing. **Samin Shiati**: Data curation; Investigation; Project administration; Writing—original draft; Writing—review & editing. **Asal Seifaee**: Data curation; Writing—original draft; Writing—review & editing. **Mohammad Sayari**: Formal analysis; Software. **Faranak Seyyedi**: Conceptualization; Investigation; Supervision; Validation; Visualization; Writing—review & editing. **Kamran Bagheri Lankarani**: Conceptualization; Methodology; Project administration; Supervision; Writing—original draft; Writing—review & editing. **Sulmaz Ghahramani**: Conceptualization; Investigation; Methodology; Project administration; Supervision; Writing—review & editing.

## ETHICAL APPROVAL

All patients gave written informed consent before the study. The Shiraz University ethics committee approved the study protocol (ethics code: IR. SUMS. REC.1400.828). Participants declared their agreement in the written informed consent for publication.

## TRANSPARENCY STATEMENT

The lead author Sulmaz Ghahramani affirms that this manuscript is an honest, accurate, and transparent account of the study being reported; that no important aspects of the study have been omitted; and that any discrepancies from the study as planned (and, if relevant, registered) have been explained.

## Data Availability

The datasets used and/or analyzed during the current study are available in figshare repository via the DOI: 10.6084/m9. figshare.23291780.
